# Catch me if you can: *Arabidopsis thaliana* lags in adaptation to contemporary climate change

**DOI:** 10.64898/2026.06.02.729671

**Published:** 2026-06-03

**Authors:** Laura Leventhal, Moises Exposito-Alonso

**Affiliations:** 1 Department of Biology, Stanford University, Stanford, CA 94305, USA.; 2 Department of Integrative Biology, University of California Berkeley, Berkeley, CA 94720, USA.; 3 Innovative Genomics Institute, Berkeley, CA 94720, USA.; 4 Howard Hughes Medical Institute, Berkeley, CA 94720, USA.

## Abstract

Anthropogenic climate change fosters unprecedented temperature challenges, with each year breaking a temperature record. Through evolution by natural selection, species and their populations have adapted to their previously local environments. However, as the average global land temperature has increased by ~2 °C or more, natural selection on many species may not act fast enough with climate change, creating an adaptation lag. To understand potential adaptation lags to recent climate change, we conducted a meta-analysis on the largest set of single-species field transplantation experiments across climates with the broadly-distributed model plant, *Arabidopsis thaliana*, with a total of 1,600 germplasm and 42 field trials. We developed a Gaussian fitness model dependent on local environment and climate deviations to infer genotype-specific adaptation lag parameters. We estimate a mean thermal adaptation lag over 1.91 °C, suggesting that local populations, on average, are better adapted when transplanted to locations cooler than their home climates. While a less than 2 °C temperature mismatch appears small, its impact on fitness corresponds to a 14% cumulative burden over time, which compounds depending on the future climate emission scenario. Combining climate model projections under different scenarios, we found that by 2025, populations would have lost 30% demographic potential under a moderate emissions scenario. Our discovery of this adaptation lag shows that even this highly adaptable species has not kept pace with recent climate change.

Global mean temperatures have already increased by ~2 °C, substantially threatening biological diversity (Exposito-Alonso et al., 2022; Habibullah et al., 2022; National Climatic Data Center, 2025). Scientists have previously documented species responses to warming through migration: by shifting their geographic ranges by 6.1 km per decade poleward and 29 m per decade in altitude (Lenoir et al., 2008; Parmesan & Yohe, 2003; Ramírez-Barahona et al., 2025) or through phenological strategies: by advancing the timing of key adaptive life-history events, such as flowering 2.3 days earlier per decade (Parmesan, 2006; Parmesan & Yohe, 2003; Wiens, 2016). However, whether populations are evolving and adapting to the new environmental conditions, or whether they are already mismatched and lagging behind climate change, remains poorly understood. Here we introduce a framework to estimate adaptation lag using large-scale common garden experiments across climates in plants in combination with climate deviations of the last decades for inference.

\ The first studies investigating adaptation to different climates in the early 20^th^ century asked whether populations within a species harbor genetic adaptations to different local environments with the knowledge that plants often experience a range of environments within their distribution (Clausen et al., 1941; Turesson, 1922). Turesson in Sweden, and Clausen, Keck, and Hiesey in California, tested this idea by collecting plants of the same species along altitudinal or latitudinal gradients, and planted them reciprocally in common gardens, quantitatively showing local adaptation: populations harbor local advantage measured by increased fitness in gardens near their locations of origin. This pattern has been replicated in hundreds of species (Hereford, 2009), and combining genome sequencing has confirmed such local adaptation has a genetic basis (Expósito-Alonso et al., 2019; Fournier-Level et al., 2011; Savolainen et al., 2013). Given its prevalence in ecology and evolution research, local adaptation data has accumulated and a recent meta-analysis showed that with just 2 °C of warming, the fitness advantage conferred by local adaptation was lost, functionally erasing the signal of local adaptation (Bontrager et al., 2020), warning that an adaptation lag may have already occurred.

We suspected that an adaptation lag to global warming may be already detectable in some species, although we expect the statistical signal across a species to be weak and challenging to infer, as many species have broad geographic ranges spanning many degrees Celsius in annual average temperature. Still, we aimed to quantify lag in the model plant *Arabidopsis thaliana*, whose geographic range spans most temperate regions across continents, and where common gardens have been deployed across dozens of locations worldwide (Leventhal et al., 2025). In total, we synthesize fitness data from up to 1614 *A. thaliana* populations transplanted across 42 experimental gardens (Fig. 1, Table S1–S2). Seeds from most of these populations (i.e. genotypes) were collected in the 90s and early 2000s (although the year of collection range for our data set is 1937–2012) from known GPS locations and maintained in seed stocks. In this meta-analysis, these seed stocks function as pseudo-”resurrection” plants relative to their collection year (Franks et al., 2007, 2018). Because the accessions were grown in controlled environments after collection, they are essentially frozen in evolutionary time since there was a no-selection environment and the accumulation of new mutations is low per generation and likely neutral. These banked genotypes were maintained by several organizations and then were often re-used in multiple common gardens, most of which were conducted during the 2010s-2020s, a time period which experienced substantial climate deviations (Fig. 1C, climate TerraClim (Abatzoglou et al., 2018)).

With this dataset in hand, we built a Gaussian fitness model under an assumption of local adaptation: each population is expected to achieve maximal fitness when the temperature of the common garden matches the temperature of its home site. We define the temperature mismatch as $d=T_{s}-T_{g}$, where $T_{s}$ is the mean annual temperature of the common garden site, $T_{g}$ is the mean annual temperature at the genotype’s location of origin in the year it was collected. With increasing distance, fitness follows Gaussian function decay, such that fitness is maximized at d=0 and declines as |d| increases. Across species, fitness responses to temperature often take a Gaussian form, as there is often a genotype or population specific optimal temperature (Latimer et al., 2015; Wooliver et al., 2022). If populations were lagging behind their optima, then the environmental transplant distance is further increased by a λ lag: $w_{\mathrm{observed}}=w_{max}exp(-(d+\lambda {)}^{2}/2V_{s})$; which would be visually a left-shifted Gaussian curve (Fig. 1C).

We estimated the parameters of our Gaussian function by transforming this function into a log quadratic regression fitted within a Bayesian hierarchical framework to account for various random effects (see **Methods** and full derivation, symbol definition Table S3, model terms Table S4, raw fitness data Fig. S1, and model diagnostics Fig. S3–4–5–6), with λ=-1.91°C (95% CI: 0.99–3.18) (Fig. 2A). The Gaussian curve decay is broad, $V_{s}\;=\;824$ (95 % CI [698, 1004]), where $V_{s}\;=\;824$ is the variance parameter of the Gaussian fitness function (i.e., the squared width of the fitness peak, such that larger values correspond to weaker stabilizing selection and slower fitness decline away from the optimum). This large value indicates a shallow fitness gradient, corroborating the common knowledge that *A. thaliana* has a broad environmental distribution; accordingly, the per-year fitness cost of a 1 °C temperature deviation from the optimum is small (~0.1 %). However, the effect of even a small deviation from the optimum can be catastrophic for a small annual plant in which fitness compounds multiplicatively through generations, because even small per-generation fitness reductions are applied to population growth each generation, leading to cumulative declines over time (Fig. S7). This is consistent with patterns shown previously in an *A. thaliana* experiment spanning four locations in Europe, where in the coldest common garden location of Oulu (Finland), accessions several degrees south in latitude had the highest fitness, not the closest local population (Wilczek et al., 2014). Our model showed that individual experiments, inducing the original lag experiment or any single study analyzed independently, did not have sufficient power to detect lag (see drop-one-out and leave-one-in re-analyses in Supplemental Materials, Table S8). In contrast, the accumulation of experiments over recent decades, particularly the addition of over 30 locations from a large-scale experimental evolution project, provided the power necessary to infer an adaptation lag.

The experimental gardens and populations included in this meta-analysis differ in many details of design and sampling, and this heterogeneity both bolsters the robustness of our species-wide mean adaptation lag estimate and provides an opportunity to examine how garden design and population attributes influence lag. Using the large sample size of our dataset (~30,000 observations), we conducted a series of exploratory re-analyses in which we re-fit the model to subsets defined by garden characteristics and accession traits (Fig. 2C, Table S7. Gardens located outside the native range of *A. thaliana* exhibited positive adaptation lags on average), consistent with the idea that non-native or invasive populations can confer novel environmental advantages that decouple populations from their historical climate optima (Callaway & Ridenour, 2004; Corbin & D’Antonio, 2010; Davidson et al., 2011). In contrast, several experimental design elements did not alter the sign of lag: whether plants were established as seeds versus seedlings or whether they were planted directly in the ground versus in pots, all subsets showed negative lag. However, pot-grown plants tended to show slightly less negative lags than ground-planted plants, suggesting that pots may buffer individuals from the full fitness costs of warming. Likewise, lag estimates from seeds versus seedling experiments were nearly indistinguishable, implying that temperature deviations in these studies did not differentially select on early life stage versus later establishment (Akiyama & Ågren, 2014; Schupp, 1995). Finally gardens that included water addition treatments produced lag estimates with notably broader confidence intervals than those without water addition, hinting at an interaction between temperature and moisture regimes that could modulate the expression of adaptation lag (Fréjaville et al., 2020; Morison & Morecroft, 2006). At the population level, most accession level groupings showed confidence intervals that crossed 0 and therefore were not contributing to significant estimates of lag. However, we observed suggestive trends; accessions collected outside the native range tended to show positive lags, relict populations from the southern glacial refugia had slightly less negative lags than non-relicts, and lag direction did not depend on the year of collection.

We then asked whether different populations had different levels of adaptation lag, how differences were distributed across space, and what are potential consequences of spatially structured adaptation lag. Our modeling approach and repeatability of populations being transplanted to multiple environments permitted us to generate genotype random effect estimates and therefore infer population-specific lag ($\lambda_{i}$) (see Supplemental Methods). Ninety-three percent of populations exhibited lag less than 0°C, and we found some variation in each population’s lag, which ranged from ±3.15 °C around the mean (i.e., populations ranged from −5.06 °C to 1.24 °C) (Fig. 2B). The degree of lag was significantly correlated with latitude (*R*^*2*^ = 0.18, P = 3.54 × 10^−14^), where lower latitude populations had more negative lag values consistent with other measure of lag in plants across a latitudinal cline (Fréjaville et al., 2020).

Such extreme levels of adaptation lag in these high temperature regions could lead to a heightened fitness burden accumulating over years of unrealized fitness potential. Plant biomass is expected to suffer a loss that varies based on the future emission scenarios. In a conservative emission scenario (RCP4.5) biomass loss is predicted to range from 6.8–12%, while in a high emissions scenario (RCP 8.5) biomass loss ranges from 13.3–20.1% (Uribe et al., 2023). In *A. thaliana,* climate change has created relatively consistent warming across the range with 982 populations showing a mean annual-temperature deviation trend from TerraClim of +0.40 °C per decade 2000–2025 that is essentially identical across latitudinal bands (Fig. 3). Although such temperature deviations appear small, they translate into substantial fitness losses when mapped through the lag model. Rearranging the Gaussian fitness function, $decline(\Delta T)=1-exp(-{(\Delta T+\mu_{2})}^{2}/(2V_{s}))$, we estimate mean fitness declines of −24.6% [−48.0,−12.1], −25.2% [−46.5,−14.2], −27.2% [−49.0,−13.6], and −29.4% [−54.6,−12.9] under SSP1–2.6 (sustainability scenario), SSP2–4.5, SSP3–7.0, and SSP5–8.5 (fossil fuel centered scenario) respectively. These projected losses are in addition to the unmeasured fitness consequences of climate lag accumulated over the previous century. To better understand the potential impact of future emission scenarios on species’ adaptation and fitness, we extracted per-year climate projections (Thrasher et al., 2022) from global circulation models (GCMs) from present until 2050 that include four shared socioeconomic pathways, SSP1–2.6, SSP2–4.5, SSP3–7.0, and SSP5–8.5, spanning sustainable development, middle-of-the-road, regional rivalry, and fossil-fueled growth futures. NASA’s NEX-GDDP-CMIP6 ensemble mean deviations at 2050 vary from +2.59 °C under SSP1–2.6 to +3.21 °C under SSP5–8.5, with SSP2–4.5 (+2.85 °C) and SSP3–7.0 (+2.89 °C) in between. In addition, individual GCM × pixel year-to-year variability is ±0.5 °C, creating substantial deviations. Using the species-wide λ, mean per-year fitness at 2050 falls between −1.69 % (SSP1–2.6) and −2.23 % declines in fitness every year (SSP5–8.5), and a total reproductive deficit accumulated from 2000 to 2050 of −36.8% (SSP1–2.6) to −40.0% fitness (SSP5–8.5) (Fig. 3B).

Finally, to directly attribute fitness deficit to future policy decisions, we compared the fitness in SSP1–2.6 (sustainability) and SSP5–8.5 (fossil fuel) scenarios. We found that failing to mitigate climate change based on Paris Agreement policies, erodes a median of −7.5% of fitness (10–90 percentile: −12.4 %, +0.8 %) by 2050 in addition to the already-large scenario of low emission/sustainability. While not the most ambitious sustainable scenario, SSP1–2.6 aims to limit global warming below 2 °C by 2100 by cutting emissions near zero by 2075 (SSP1–1.9 not included); although this scenario has already been rendered unlikely given the failing of policy commitments based on United Nations Environment Programme (UNEP) assessments and climate policy tracking (United Nations Environment Programme, 2025) (Fig. 3C). We cannot permit reaching the worst-case scenario, and we must re-uphold policies to at least an intermediate SSP2–4.5 scenario, which would nevertheless mean accepting a loss of −25.2% in fitness (Fig. 3C SSP4.5).

Our observations are consistent with a growing body of literature suggesting that adaptation lag may be more prevalent across plant species than previously appreciated. For example, in the Rock Mountains, a closely related mustard, *Boechera stricta*, had populations that appear unable to track earlier snowmelt through upslope migration, leading to maladaptation and motivating efforts toward genetic rescue (Anderson et al., 2025). Similarly, *Quercus lobata* populations in California have been shown to fall outside their historical climate niche (Browne et al., 2019). Applying our adaptation lag model, we extend these findings by quantitatively estimating evolutionary mismatch across species using publicly available transplant experiments (Table 1). Across 17 vascular plant species with diverse experimental designs, we were able to detect significant adaptation lags in four species using different fitness metrics. The perennial switchgrass (*Panicum virgatum*) showed −6.1 °C [−5.3, −6.8], the maritime pine (*Pinus pinaster*) at −6.0 °C, and the aforementioned Valley Oak (*Quercus lobata*) showed a clear lag but optimum could not be inferred because all gardens sit entirely on one flank of the thermal optimum, indicating at least a ≥ −6–8 °C maladaptation lag. Only one species, Atlantic white cedar (*Chamaecyparis thyoides*) yielded a reversed lag of +1.3 °C [+2.5, +0.1] (i.e. populations performed better in warmer environments), probably because only three gardens were assessed and the southernmost garden permitted winter survival independent on population of origin. We believe most species do not yet have sufficient data to yield significant results. A joint model across all species excluding *A. thaliana* with normalized fitness and taxon and study as random effect shows an average adaptation lag, although non-significant (−0.73°C [CI95% −4.36, +1.49], 75% of posterior distribution indicates lags). Even with statistical uncertainty and species-to-species heterogeneity, having such a lag across species would mean a massive impact on fitness of plant species worldwide in the next decades. Even under middle-of-the-road scenario, by mid-century any given year’s deviations will surpass the uncertainty threshold across such non-model species (SSP2–4.6, 2025 ${\Delta T}_{land}\;=\;1.3{}^{\circ}C$), which we may estimate back-of-the envelope fitness loss of 1.8% fitness (CI95% 0.01% - 23.4%) (Table S5). The fossil-fuel-favoring socioeconomic scenario (SSP5–8.5, 2025 ${\Delta T}_{land}\;=\;+2.1{}^{\circ}C$) would imply a 3.5% fitness loss (0.06%−29.4%) (Table S5). Some species with the largest lags such as perennial bunchgrass, Pines, or Valley Oaks, are expected to have a cumulative fitness loss of 10 %, 22%, and 60%, respectively, in the optimistic scenario (SSP2–4.6) (Table S5). Because these impacts propagate across populations and across species, the compound fitness loss of ecosystems would be insurmountable (Table S6).

Much work and literature on the ability of plants to adapt to climate change focuses on if there is enough standing genetic variation to enable adaptation (Barrett & Schluter, 2008). This analysis suggests that at least for a model system of relatively low conservation concern, standing genetic variation is not enough to adapt to climate change. Measuring adaptation lag across species will be essential for communicating with policy makers and predicting which species or populations are most at risk. While it is imperative to understand the extent of adaptation lag across species on Earth, in order to approach biology from a place of curiosity and wonder rather than urgency, the only true imperative is to slow the rate of global climate change.

## Supplementary Material

Supplement 1

## Figures and Tables

**Figure 1.**
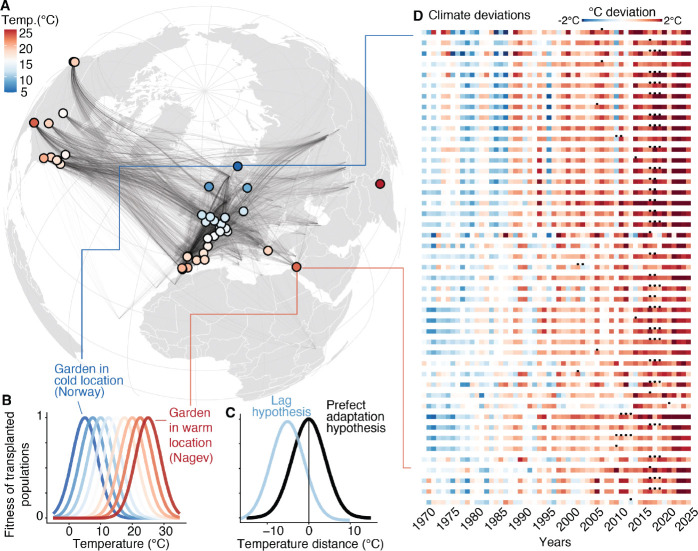
42 transplant common gardens and climate deviations for meta-analysis of adaptation lag. (A) Global projection of common gardens used in this study and the population (or accessions) that were planted in each location. Points represent the garden locations and are colored by temperature, while black lines represent accessions. The lines start at the population’s home coordinates and end in the garden(s) in which they were planted. (B) Representation of local adaptation with Gaussian fitness curves, where the fitness maximum is reached when the temperature of the site matches the home temperature of the genotype. (C) Adaptation lag visualization, where if fitness maxima is reached at no temperature distance, then no adaptation lag occurs. A left shifted fitness peak suggests adaptation lag consistent with climate change predictions, where a genotype is adapted to an environment cooler than their current environment. (D) Diagram of climate deviations by experimental site use in this meta analysis. Gardens are sorted by latitude, and points on the bars represent the year the experiment was conducted in that location. The climate deviations were calculated by subtracting the Terraclim temperature value from the given year by the mean baseline annual temperature for that area (1970–2000). Deviations are all relative to that garden location's specific baseline.

**Figure 2.**
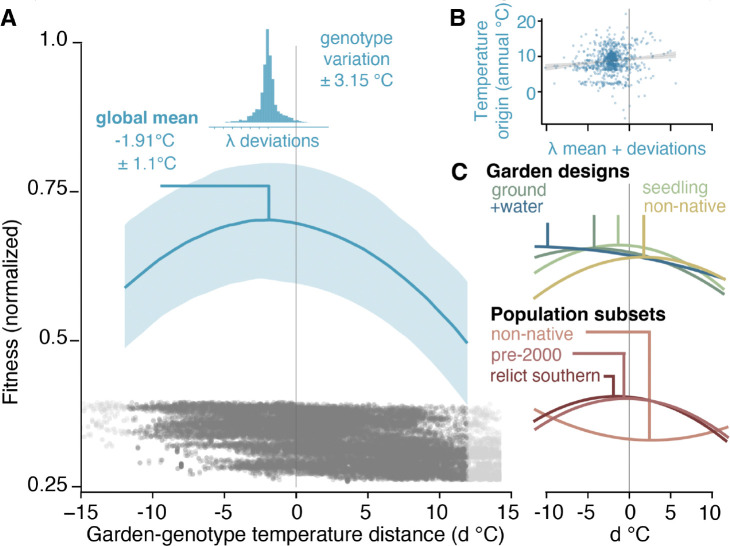
Estimate of global adaptation lag across *Arabidopsis thaliana*. (A) Mean adaptation lag for accessions in our meta analysis is at −1.91 °C, suggesting adaptation to cooler environments. (B) Spread of adaptation lag across genotypes. X-axis is genotype-level adaptation lag minus mean adaptation of all genotypes, showing mean lag therefore centered at zero. (C) Curiosity tests of different subsets of the data on adaptation lag. We grouped our data set by garden design parameters (top) such as if the plants starting as seeds or seedlings outside, were planted in the ground vs. pots, had a water addition treatment, and finally if the garden was located in the non-native *A. thaliana* range. We also investigated population-specific groupings (bottom) such as if the population was collected before the year 2000, was collected non-native range, or is considered a relict (ice age refugia survivors).

**Figure 3.**
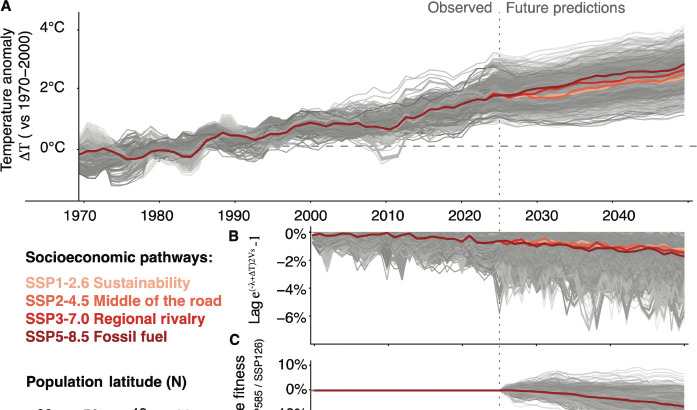
Projections of future adaptation lags under socio-economic global change scenarios. (A) Observed climate deviations 1970–2025 observed at each *Arabidopsis thaliana* population colored by latitude (source: TerraClim). Deviations are calculated for every year with respect to the historical 1970–2000 average (Worldclim). Future climate change predictions start in 2026 using 5 year moving averages. (B) Population fitness loss relative to the optimum estimated from transplants due to measured adaptation lag (λ = −1.91 °C) and ongoing temperature deviations (ΔT). In the future, multiple lines per location are plotted and thick lines represent the average across socioeconomic pathways scenarios. (C) Difference in cumulative fitness loss between a sustainable scenario (SSP1–2.6) and fossil fuel development (SSP5.8.5).

**Table 1 |:** Plant common-garden panel to re-fitting the climate-lag model.

Species	N gardens	N sources	Cov. (%)	d_range_ (°C)	*V*_s_ [CI95] (°C^2^)	μλ [CI95] (°C)

***Arabidopsis thaliana***	45	1,564	207%	43.1	814.7 [716.8, 968.9]	**−1.9 [−2.9–1.1]**
***Quercus lobata***	2	658	—	4.6	n/a (β_2_ > 0)	**≥ −6 to −8**
***Panicum virgatum***	10	592	67%	—	109.7 [103.0, 116.9]	**−6.1 [−6.8, −5.3]**
*Fagus sylvatica*	29	202	71%	12.7	1,057 [335, 11,944]	−2.6 [−61.8,+6.3]
*Picea glauca*	16	242	51%	19.5	69.8 [52.1, 105.3]	+0.8 [−0.4, +1.8]
*Plantago lanceolata*	16	38	78%	29.6	353 [139, 5,763]	+4.3 [−3.8, +51]
*Juglans nigra*	7	66	66%	12.6	44 [23, 188]	−0.5 [−4.1,+4.9]
*Silene flos-cuculi*	18	22	42%	13.6	69 [22, 310]	+6.5 [−3.3,+35]
*Chamaecrista fasciculata*	16	19	48%	23.4	127 [50, 1,321]	+3.2 [−1.6, +8.3]
*Boechera stricta*	5	52	22%	4.4	8.4 [4.2, 47.5]	+1.5 [−0.0,+3.2]
*Lotus corniculatus*	7	32	21%	5.0	8 [3, 75]	+1.4 [+1.2, −5.9]
*Populus tremuloides*	5	43	23%	7.4	27 [15, 94]	+0.5 [−1.3, +2.7]
***Pinus pinaster***	5	34	56%	10.9	243.7 [104.9, 2,299.2]	**−6.0 [−51.7,−0.9]**
***Chamaecyparis thyoides***	3	34	96%	18.8	56 [42, 86]	**+1.3 [+0.1, +2.5]**
*Arabidopsis lyrata*	7	12	33%	18.5	127.3 [43.1, 1,120.4]	+3.7 [−7.5, +36.2]
*Pinus canariensis*	4	21	88%	11.9	155 [44, 1,466]	−5.0 [−28, +2.2]
*Erythranthe guttata*	4	14	46%	10.9	14 [7, 92]	−2.2 [−8.2, +0.1]

**Notes.** *Q. lobata* μ_λ_ is a directional lower bound (gardens don’t bracket the Gaussian peak); design power counts the 2 common gardens × 5 blocks each. *A. thaliana*’s source coverage exceeds 100% because the pipeline uses per-year TerraClimate annual T at source locations (with cold-year deviations down to −11.7 °C) while GBIF coverage is computed on the 1970-2000 climatology. **Species-level climate source**. GBIF retrieved 2026-04-20 via rgbif::occ_search (hasCoordinate = TRUE, hasGeospatialIssue = FALSE, limit 1,500). Bio1 overlay: WorldClim v2.1 10-arcmin.

Species sorted by design power (N gardens × N source populations). Source coverage = fraction of the species’ native mean-annual-temperature range (from up to 1,500 GBIF occurrences per species, sampled on WorldClim v2.1 bio1) spanned by the study’s source populations. *d* range is the experimental *d*_temp_ = garden − source climate difference span. Gaussian-lag parameters *V*_*s*_ (niche width,C °C^2^) and (climate lag, °C — positive = adapted to cooler than current home) are from independent per-species MCMCglmm fits with no prior, using year-specific site climate. Values in **bold** have 95% credible intervals that exclude zero.

## Data Availability

All scripts are available at Zenodo: <DOI add>.
