# Supplementary material for: Catch me if you can: Arabidopsis thaliana lags in adaptation to contemporary climate change
Source: bioRxiv. 2026 Jun 3:2026.06.02.729671. Preprint. [Version 1] doi: 10.64898/2026.06.02.729671 (PMC13252057; doi:10.64898/2026.06.02.729671)
Supplement: Supplement 1 [file NIHPP2026.06.02.729671v1-supplement-1.pdf]

bioRxiv preprint doi: https://doi.org/10.64898/2026.06.02.729671; this version posted June 3, 2026. The copyright holder for this preprint (which was not certified by peer review) is the author/funder, who has granted bioRxiv a license to display the preprint in perpetuity. It is made available under a CC-BY 4.0 International license.

Supplementary Materials

Table of content

Supplementary Materials **11**
Methods and Supplemental Tables 13
Data collection 13
Table S1. Summary of common garden experiments included in meta-analysis. 13
Fitness data 13
Table S2. Fitness metrics chosen from each common garden study 14
Climate data sources 14
Historical observations (1970–2025) 14
Future projections (2026–2050) 15
Per-GCM bias correction 15
Temperature deviations 15
Visual smoothing 15
Modeling adaptation lag 16
Table S3. Mathematical symbols used in adaptation lag model 16
Table S4. Mapping between mixed-model parameters and Gaussian lag parameters. 18
Fitness projections into future climates 18
Gaussian lag model 18
Two lag variants 19
Per-year relative fitness 19
Cumulative fitness shortfall under the worst-case scenario 19
Posterior sampling of the lag 20
Average species loss 20
Table S4. Potential fitness loss in different climate change socioeconomic scenarios. 20
Table S5. Potential fitness losses of each species. 21
Table S6. Cumulative aggregate of fitness decline (2050 land warming) 21
Table S7. Curiosity testing lag measurements based on accession or garden characteristics. 22
Table S8. Sensitivity testing, leave one out and keep one in results. 22
Supplemental Figures **23**
Figure S1. Relationship between fitness and annual temperature mismatch between experimental and genotype home site. 23
Figure S2. Climate space of common garden sites and genotypes. 24
Figure S3. Model diagnostics for fixed effects in the adaptation-lag model. 25
Figure S4. MCMC diagnostics for variance components in the adaptation-lag model. 26
Figure S5. Heteroscedasticity diagnostics for the adaptation-lag model. 27
Figure S6. Heteroscedasticity diagnostics showing per-garden distribution of residuals from the

bioRxiv preprint doi: https://doi.org/10.64898/2026.06.02.729671; this version posted June 3, 2026. The copyright holder for this preprint (which was not certified by peer review) is the author/funder, who has granted bioRxiv a license to display the preprint in perpetuity. It is made available under a CC-BY 4.0 International license.

adaptation-lag model. 28
Figure S7. Posterior distributions for key adaptation-lag parameters. 29

---

bioRxiv preprint doi: https://doi.org/10.64898/2026.06.02.729671; this version posted June 3, 2026. The copyright holder for this preprint (which was not certified by peer review) is the author/funder, who has granted bioRxiv a license to display the preprint in perpetuity. It is made available under a CC-BY 4.0 International license.

Methods and Supplemental Tables

Data collection

We searched for outdoor common garden experiments (73 studies total), many of which we had already documented in (Leventhal, Ruffley, and Exposito-Alonso 2025). Studies were published between 1993 and 2021 and mostly occurred in the northern hemisphere. We selected studies that used natural accessions (no mutant plants, mutation accumulation lines, or recombinant inbred lines), and most accessions were from the 1001 Genomes and RegMap, while some were wild accessions not in either resource. If a study was from an outdoor common garden and (1) contained more than one natural accessions and (2) recorded a component of fitness (survival, silique number, fruit length, seed count, seed weight, rosette diameter, inflorescence height, number of branches), we included that study in our analysis. Because it is naturalized globally, we included both accessions and gardens outside of the native range, although we have a column noting this in our data set to use a co-variate (see below). In total our data came from 10 studies spanning 48 gardens (**Table S1**) with experiments conducted between the years 2002 to 2021.

Table S1. Summary of common garden experiments included in meta-analysis.

Author	Year	Number of accessions	Accession origin	Pots/ground	Seeds/seedlings
Brachi	2015	398	REG map	pots	seedlings
Exposito-Alonso	2018	50	Wild	pots	seeds
Exposito-Alonso	2019	517	1001 Genomes	pots	seeds
Korves	2007	169	ABRC	ground	seedlings
Leventhal	2025	245	1001 Genomes	pots	seeds
Manzano-Piedras	2014	279	Wild	pots	seeds
Rutter	2007	21	Wild and 1001 Genomes	ground	seedlings
Singh	2017	45	Wild	pots	seeds
Wilzcek	2014	230	REG map	ground	seedlings
Wu	2026	231	1001 Genomes	pots	seeds

Fitness data

Across all fitness metrics, we had 78,856 observations across 1,655 distinct accessions. This includes a measurement for every accessions across the permutations of experiment (an individual publication), site (the experimental location), year (the year the garden was planted), season (the time of year the garden was planted, namely for Fall and Spring), and the treatment, which only applied to two of the studies. When we filtered the data to fecundity and lifetime fitness, there were 28,853 observations across 1614 distinct accessions. Some studies provided a measure of lifetime fitness (essentially survival × fecundity), but some studies would measure both survival and some reproductive output (e.g., fruit number or seed weight). To model lifetime fitness in the case of both survival and fecundity being provided, we modified these studies to have a lifetime fitness measurement by multiplying survival by fecundity. The fitness metrics that were used in the model for each study is included in **Table S4**.

The column is log-normalized within each garden ($logW_{ijk} - \underline{logW}_k$). We apply a further within-garden min–max rescaling using the 1st–99th percentile as robust anchors, mapping every garden to the interval $[0, 1]$. This preserves rank order and makes V_s comparable across studies.

bioRxiv preprint doi: https://doi.org/10.64898/2026.06.02.729671; this version posted June 3, 2026. The copyright holder for this preprint (which was not certified by peer review) is the author/funder, who has granted bioRxiv a license to display the preprint in perpetuity. It is made available under a CC-BY 4.0 International license.

Table S2. Fitness metrics chosen from each common garden study

Study	Selected fitness metric	Rationale
Brachi 2015	Lifetime fitness	Pre-computed metric in original study (total length of mature fruits produced by each plant)
Exposito-Alonso 2018	Survival × fruits	Mean number of rosettes (mean maximum number of rosettes per block) × Fruits (mean per reproductive plant)
Exposito-Alonso 2019	Fitness	Pre-computed combined metric; each Density × Water combination is a distinct garden environment
Korves 2007	Total seed mass × survival for winter survivors	Mean total seed mass per accession × Survival
Leventhal 2025	Lifetime fitness	Survival (proportion, 0-1) × Fruits (mean per surviving plant); each precipitation treatment is a distinct garden
Manzano-Piedras 2014	Rosettes × fruits	Pre-computed combined metric; single garden but genotypes overlap heavily with other studies
Rutter 2007	Fruits	Pre-computed combined metric
Singh 2017	Number of fruits	Pre-computed combined metric
Wilzcek 2014	Seed number	Pre-computed fitness metric
Wu 2026	Accession frequency	Integrates survival + fecundity + competition over 3 years × 31 gardens

Climate data sources

We recorded the latitude and longitude of each experimental site from the published manuscripts, and if coordinates were not given, the closest possible location was selected. We collected the latitude and longitude for each accession from either the home database (1001 Genomes or RegBank) or in the case of wild accessions, from the respective manuscript. For the experimental locations climate data, we gather the year of the experiment and coordinates, and pulled the corresponding data from TerraClim. For the accessions' climate data, we gathered the year the accession was collected from the 1001 Genomes or RegBank database or from the records of the authors in the case of wild accessions. If a range of years of collection was given for wild accessions, we selected the median year for those accessions. If the year of collection was not provided in the 1001 Genomes database or RegBank database, the median year of collection for all accessions was used. In the case that the collector of an accession was known but the year was not known, we used the median year for all accessions collected by that individual. Once every accession had a recorded year of collection, using TerraClim we pulled the climate for that year at the given accession's coordinates. 4.7% (77/1617) of the accession were collected before 1958 (the earliest year of TerraClim data), then the year 1958 was used. From TerraClim, we selected monthly mean temperature (°C), monthly cumulative precipitation (mm), and the coefficient of variation in monthly temperature (higher values means more space in between rainfall events). We then calculated mean annual temperature (°C), annual precipitation accumulation (mm), and mean annual monthly coefficient of variation in precipitation.

Historical observations (1970–2025)

We extracted annual mean air temperature at every focal pixel from **TerraClimate** v1.5 (Abatzoglou et al. 2018), a 1/24° (≈ 4 km) global monthly reanalysis of minimum and maximum surface air temperature. For each pixel and calendar year we took the twelve monthly values of $\left(T_{min} + T_{max}\right)/2$ and averaged

bioRxiv preprint doi: https://doi.org/10.64898/2026.06.02.729671; this version posted June 3, 2026. The copyright holder for this preprint (which was not certified by peer review) is the author/funder, who has granted bioRxiv a license to display the preprint in perpetuity. It is made available under a CC-BY 4.0 International license.

them to a single annual mean $\overline{T}_y$ covering 1970–2025. TerraClimate NetCDF files were downloaded in advance from the Climatology Lab and read locally with the **terra** R package.

Future projections (2026–2050)

Future air temperature came from the NASA NEX-GDDP-CMIP6 bias-corrected statistically downscaled ensemble (Thrasher et al. 2022), which provides daily mean near-surface air temperature (tas) on a 0.25° grid. We fetched all four standard Shared Socioeconomic Pathway scenarios spanning the emissions spectrum: SSP1-2.6 (sustainability, low emissions), SSP2-4.5 (middle of the road), SSP3-7.0 (regional rivalry, high emissions), and SSP5-8.5 (fossil-fuelled development). For every scenario we ran a five-GCM ensemble: ACCESS-CM2, EC-Earth3, MPI-ESM1-2-HR, MRI-ESM2-0, and UKESM1-0-LL, all at the r1i1p1f1 variant on each model's native grid (except UKESM1-0-LL, which uses r1i1p1f2). NEX-GDDP daily files were retrieved one year at a time from the NASA Center for Climate Simulation THREDDS server (https://ds.nccs.nasa.gov/thredds/fileServer/AMES/NEX/GDDP-CMIP6/...), with incremental appends to a single cached CSV so the download is resumable. Daily values were averaged over each year to give an annual mean temperature per (pixel, scenario, GCM, year).

Cite NASA (10)

Per-GCM bias correction

TerraClimate (4 km, downscaled from CRU/WorldClim) and NEX-GDDP (25 km, bias-corrected against GMFD/ERA5) differ in absolute temperature scale by 0.5–2 °C in topographically complex regions. To make the two series directly stitchable we applied a per-GCM, per-pixel delta-method correction over the 2015–2025 overlap:

$$\hat{T}_{g,p,y} = T^{NEX}_{g,p,y} + \left[\overline{T}^{TC}_{p,2015-2025} - \overline{T}^{NEX,\,SSP2-4.5}_{g,p,2015-2025}\right],$$

where g indexes GCM, p the 0.25° pixel, and y the year. The offset is computed from SSP2-4.5 only and reused across every scenario, because all four SSPs share almost identical forcings over 2015–2025 (we verified empirically that each GCM's 2015–2025 mean varies by ≤ 0.3 °C across scenarios). Using a single-scenario overlap keeps every SSP on a common TerraClimate-anchored scale and preserves the relative differences between SSPs exactly.

Temperature deviations

Per-pixel deviations are expressed against a 1970–2000 TerraClimate baseline:

$$\Delta T_{p,y} = T^{\star}_{p,y} - \overline{T}^{TC}_{p,1970-2000},$$

with $T^{\star}_{p,y}$ equal to TerraClimate's annual mean for $y \leq 2025$ and to the bias-corrected NEX-GDDP value $\hat{T}_{g,p,y}$ for $y \geq 2026$. TerraClimate observations 1970–2025 are identical across GCMs and scenarios and are replicated through every (scenario, GCM) channel so each projection trajectory is continuous from 1970 to 2050.

Visual smoothing

Annual values have ~0.4–0.5 °C year-to-year internal variability per GCM, which swamps the ~0.6 °C scenario divergence at 2050. For display we apply two sequential operations before plotting:

1. **GCM collapse**: for each (pixel, scenario, year) take the mean across the 5 GCMs. This removes model-internal variability from the display while preserving inter-scenario contrast.
2. **Five-year centred rolling mean** (`stats::filter` with `rep(1/5, 5)`, `sides = 2`) on each resulting per-(pixel × scenario) series. Smoothing is applied *after* the GCM collapse so it reduces noise without distorting ensemble means.

bioRxiv preprint doi: https://doi.org/10.64898/2026.06.02.729671; this version posted June 3, 2026. The copyright holder for this preprint (which was not certified by peer review) is the author/funder, who has granted bioRxiv a license to display the preprint in perpetuity. It is made available under a CC-BY 4.0 International license.

Per-scenario grand means across populations (the thick grey/black lines in Panel A) are computed from the GCM-collapsed series and smoothed with the same 5-year window.

Modeling adaptation lag

Here we define the notation used in the theoretical derivation of the lag model.

Table S3. Mathematical symbols used in adaptation lag model

Symbol	Interpretation
g	Origin (home site) of genotype *i* (conceptual label
k	Experimental site (garden) index (k - 1, …, 42)
j	Replicate / individual plant within a genotype-site combination
$T_{s,k}$	Temperature at experimental site *k* (°C); *s* denotes "site"
$T_{g,i}$	Home-site temperature for genotype *i* (°C)
d_{ik}	Temperature mismatch for genotype *i* in site *k*, $d_{ik} = T_{s,k}$ - $T_{g,u}$ (°C)
λ_i	Adaptation lag for genotype *i* along the temperature axis (°C)
d^*_i	Mismatch at which genotype *i* attains peak fitness, $d^*_i = -\lambda_i$ (°C)
μ_λ	Mean adaptation lag across genotypes (°C)
$\sigma^2_{\lambda i}$	Among-genotype variance in adaptation lag (°C^2)
V_s	Width of the Gaussian fitness function (= $2\omega^2$, units °C^2)
W_{max}	Maximum relative fitness (at the optimum)
W_{ijk}	Fitness of genotype *i* in site *k*, replicate *j*
ε_{ijk}	Multiplicative error term for fitness of genotype *i* in site *k*, replicate *j*

$\lambda_i = 0$: genotype *i* is optimally adapted to its current home climate.

$\lambda_i > 0$: genotype *i* is adapted to a cooler past climate (lags behind recent warming).

$\lambda_i < 0$: genotype *i*'s optimum is shifted toward warmer conditions than its home climate.

A population is locally adapted when individuals perform best in a site whose climate matches their home climate. Under climate change, an **adaptation lag** arises when a genotype is still tuned to a *past* optimum: it reaches peak fitness not at sites matching its current home climate T_g, but at sites whose climate resembles $T_g - \lambda_i$ (a cooler past state) (visualized with raw data in **Fig. S2**). We model relative fitness as a Gaussian function of the difference between the site climate T_s and the genotype's home climate T_g: $W_{ijk} = W_{max} \cdot \exp[-(T_{s,k} - (T_{g,i} - \lambda_i))^2 / V_s] \cdot \varepsilon_{ijk}$.

To account for variations in genotype-level lag variation, we denote per genotype lag to λ_i.

$$\lambda_i \sim N\left(\mu_\lambda,\ \sigma^2_\lambda\right)$$

μ_λ: mean lag across all genotypes. σ_λ: standing genetic variation in thermal optimum.

bioRxiv preprint doi: https://doi.org/10.64898/2026.06.02.729671; this version posted June 3, 2026. The copyright holder for this preprint (which was not certified by peer review) is the author/funder, who has granted bioRxiv a license to display the preprint in perpetuity. It is made available under a CC-BY 4.0 International license.

In this Gaussian fitness function, W_{max} controls the height of the curve (maximum relative fitness), V_s controls its width (how quickly fitness declines as mismatch increases), and the term in the numerator of the exponent, $\left(T_{s,k} - \left(T_{g,i} - \lambda_i\right)\right)^2$, determines where the peak falls along the temperature axis. The lag parameter λ_i shifts the optimum along this axis, so genotypes with different λ_i values have different peak climates.

For algebraic convenience, we re-express the model in terms of temperature mismatch. If we define the climate distance as $d_{ik} = T_{s,k} - T_{g,i}$, the model becomes:

$$W_{ijk} = W_{max} exp\left(-\frac{\left(d_{ik}+\lambda_i\right)^2}{V_s}\right) \cdot \varepsilon'_{ijk}$$

When we transform this model to a log scale our full Gaussian model becomes:

$$logW_{ijk} = logW_{max} - \frac{\left(d_{ik}+\lambda_i\right)^2}{V_s} + \varepsilon'_{ijk}$$

where ε'_{ijk} is the residual error on the log scale. For notational simplicity, we write this term as ε in the derivations below.

The fitness peak in d-space is at $d^* = -\lambda_i$: a lagged genotype ($\lambda_i > 0$) reaches peak fitness at sites *cooler* than its home ($d < 0$). Because the log-Gaussian is a downward-opening parabola in d_{ik}, we can expand $\left(d_{ik} + \lambda_i\right)^2$ and rewrite the model as a quadratic function of mismatch:

$$\left(d_{ik} + \lambda_i\right)^2 = d_{ik}^2 + 2d_{ik}\lambda_i + \lambda_i^2.$$

Distributing $-1/V_s$ gives

$$logW_{ijk} = logW_{max} - \frac{\lambda_i^2}{V_s} - \frac{2\lambda_i}{V_s}d_{ik} - \frac{1}{V_s}d_{ik}^2 + \varepsilon.$$

We can recognize this as

$$logW_{ijk} = \underbrace{logW_{max} - \frac{\lambda_i^2}{V_s}}_{genotype-specific\ intercept} + \underbrace{\left(-\frac{2\lambda_i}{V_s}\right)}_{genotype\ slope\ on\ d_{ik}} d_{ik} + \underbrace{\left(-\frac{1}{V_s}\right)}_{fixed\ quadratic\ term} d_{ik}^2 + \varepsilon.$$

This suggests a random-slope mixed model with a fixed quadratic term:

$$logW_{ijk} \approx \left(\beta_0 + u_{0i}\right) + u_{1i}d_{ik} + \beta_2 d_{ik}^2 + v_{0k} + \varepsilon'_{ijk},$$

where u_{0i} and u_{1i} are genotype-specific random intercepts and slopes, β_2 is a fixed quadratic effect shared across genotypes, and v_{0k} is a garden random intercept absorbing unmeasured garden-level variation.

bioRxiv preprint doi: https://doi.org/10.64898/2026.06.02.729671; this version posted June 3, 2026. The copyright holder for this preprint (which was not certified by peer review) is the author/funder, who has granted bioRxiv a license to display the preprint in perpetuity. It is made available under a CC-BY 4.0 International license.

Table S4. Mapping between mixed-model parameters and Gaussian lag parameters.

Model term	Formula	Gaussian parameter
Fixed quadratic *on* d_{ik}^2	$\beta_2 = \frac{-1}{V_s}$	$V_s = \frac{-1}{\beta_2}$
Fixed linear on d_{ik}	$\beta_1 = \frac{-2\mu_\lambda}{V_s}$	$\mu_\lambda = \frac{-\beta_1 V_s}{2}$
Genotype random slope	$u_{1i} = \frac{-2\lambda_i}{V_s}, \quad u_{1i} \sim N(0, \frac{4\sigma_\lambda^2}{V_s^2})$	$\lambda_i = \mu_\lambda - \frac{u_{1i} V_s}{2}$
Genotype random intercept	$u_{0i} \approx \frac{-\lambda_i^2}{V_s}$	fitness cost of being off-optimum
Garden random intercept	v_{0k}	unmeasured garden-level effects

Under this reparameterization, the data identify the lag distribution through the fixed and random effects in the quadratic model. Genotype random slopes are informed by the pattern of fitness across the range of d_{ik} values observed for each genotype, and study-level intercepts absorb differences in absolute fitness among gardens.

Specifically, the model allows us to estimate (1) The **mean lag** μ_λ, which is identified by the fixed linear slope β_1 relating log fitness to temperature mismatch d_{ik}. This parameter describes the average displacement of the fitness optimum along the temperature axis across all genotypes, (2) the **standard deviation of lag** σ_λ, which is identified from the variance of the genotype-specific random slopes u_{1i} (this variance quantifies how much genotypes differ from one another in their lag, i.e. the spread of thermal optima around the mean), and (3) the **individual deviations** $\lambda_i - \mu_\lambda$ for each genotype, which are obtained as the best linear unbiased predictors (BLUPs) of the random slopes and are centered at zero by definition of the random-effects structure. These deviations show which genotypes lag more or less than the average and by how much. We do not attempt to estimate a separate W_{max} for each genotype, because fitness is rescaled within gardens and garden-level intercepts v_{0k} absorb differences in absolute fitness among experiments.

Fitness projections into future climates

Gaussian lag model

Per-year relative fitness is drawn from the Gaussian-selection reparameterisation of the Leventhal & Expósito-Alonso (2026, in prep.) MCMCglmm fit (`fit_main_10_to_10_year_fixed.rds`):

$$w_{p,y} = exp\left(-\frac{\left(\lambda+\Delta T'_{p,y}\right)^2}{V_s}\right),$$

where V_s is the species-level selection width (posterior median 824.2) and λ is the per-population lag. We re-anchored each trajectory at year 2000 so $\Delta T'_{p,y} = T_{p,y} - T_{p,2000}$. Year 2000 is the end of the 1970–2000 climatology baseline, so anchoring there keeps the fitness reference (0 %) conceptually consistent with

bioRxiv preprint doi: https://doi.org/10.64898/2026.06.02.729671; this version posted June 3, 2026. The copyright holder for this preprint (which was not certified by peer review) is the author/funder, who has granted bioRxiv a license to display the preprint in perpetuity. It is made available under a CC-BY 4.0 International license.

the temperature-deviation baseline while still letting the trajectory accumulate the historical warming observed over 2000–2025.

Relative fitness at warming ΔT above today:

$$\frac{W(\Delta T)}{W_{max}} = exp\left[-\frac{\left(\Delta T+\mu_\lambda\right)^2}{2V_s}\right]$$

Fractional decline already present at current climate ("baseline" lag cost):

$$decline_{baseline} = 1 - exp\left(-\mu_\lambda^2/\left(2V_s\right)\right)$$

Fractional decline at future warming ΔT:

$$decline(\Delta T) = 1 - exp\left(-\left(\Delta T + \mu_\lambda\right)^2/\left(2V_s\right)\right)$$

Two lag variants

We ran the same pipeline with two choices of λ:

1) **Population-specific lag (λ_i).** Each population uses its own posterior-median random-slope BLUP λ_i. The across-population spread in λ_i captures biological variation in adaptation lag.
2) **Species-average lag (μ_λ).** All populations share the species mean μ_λ (posterior median +1.96 °C), computed as $\mu_\lambda = -\beta_1 V_s/2$ from the fit's fixed effects. Using μ_λ removes biological variation from the display so any remaining across-population spread reflects only differences in home climate (through $T_{p,2000}$) and scenario/GCM uncertainty. Figure 3 uses the species-average formulation.

Per-year relative fitness

For every (population, scenario, year) we computed $w_{p,y}$ using the species-wide μ_λ draw attached to that scenario's GCM (see *Posterior sampling*). We then averaged across the 5 GCMs per (population, scenario, year) and smoothed the resulting series with a 5-year centred rolling mean, identical to the climate-data treatment. The plotted y-value is $w_{p,y} - 1$ expressed as a percentage, so 0 % corresponds to the undisturbed Gaussian optimum ($\lambda = 0, \Delta T' = 0$) and negative values indicate a yearly fitness deficit relative to that optimum.

Cumulative fitness shortfall under the worst-case scenario

Cumulative fitness from the 2000 anchor compounds the per-year values multiplicatively:

$$W_{p,Y} = \prod_{y=2000}^{Y} w_{p,y}.$$

To compare scenarios we paired each population × GCM combination and took the ratio of cumulative fitness under SSP5-8.5 (fossil-fuelled, worst-case) relative to SSP1-2.6 (sustainability, best-case):

$$R_{p,Y}^{585/126} = \frac{W_{p,Y}^{SSP585}}{W_{p,Y}^{SSP126}} - 1.$$

bioRxiv preprint doi: https://doi.org/10.64898/2026.06.02.729671; this version posted June 3, 2026. The copyright holder for this preprint (which was not certified by peer review) is the author/funder, who has granted bioRxiv a license to display the preprint in perpetuity. It is made available under a CC-BY 4.0 International license.

$R < 0$ indicates that SSP5-8.5 imposes a greater cumulative fitness cost than SSP1-2.6; $R > 0$ indicates the rare case where a population's optimum sits above its current climate and would benefit from faster warming ($\lambda_i < 0$). λ is *shared* between the two scenario sides within each (population × GCM) pair, so the ratio isolates scenario effects rather than λ-posterior noise. The ratio series is smoothed with the same 5-year rolling mean.

Posterior sampling of the lag

We propagate posterior uncertainty on the lag by drawing from a Normal approximation to its 95 % credible interval:

$$\sigma_\lambda = \frac{\lambda^{high} - \lambda^{low}}{2 \cdot 1.96}.$$

For the species-average variant used in Figure 3, μ_λ is a single scalar; we draw one posterior sample per GCM to simulate the dispersion expected due to uncertainty. Because we have many populations with variable climate trajectories, and we have multiple GCM scenarios, this gives independent samples of μ_λ capturing a natural posterior fan that is more computationally efficient without the need for time-consuming replicate loops. All populations under a given GCM share the same μ_λ draw, so the SSP5-8.5-vs-SSP1-2.6 ratio stays well-behaved under tail samples (if the two scenario sides used independent draws, extreme lag values in one direction would send the ratio to implausible values). V_s is not resampled and is fixed at its posterior median.

Average species loss

Taking *A. thaliana* aside, using a model of non-Arabidopsis species (also excluding *Quercus lobata*) we can make predictions of fitness loss in 2050.

Table S4. Potential fitness loss in different climate change socioeconomic scenarios.
Parameters: $\mu_\lambda = +0.73$ [−1.49, +4.36] °C; $V_s = 112$ [60, 324] °C² (σ_s ≈ 10.6 °C).

Scenario	ΔT (land)	Typical decline (μ_λ=+0.73)	Lower CI (μ_λ=−1.49)	Upper CI (μ_λ=+4.36)
Today ($\Delta T = 0$)	0	0.2%	1.0%	15.4%
SSP1-1.9	+0.5	**0.7%**	0.2%	17.9%
SSP1-2.6	+0.9	**1.2%**	0.05%	20.6%
SSP2-4.5	+1.3	**1.8%**	0.01%	23.4%
SSP3-7.0	+1.7	**2.6%**	0.01%	26.4%
SSP5-8.5	+2.1	**3.5%**	0.06%	29.4%

At the Model G' typical-plant level, 2050 land-warming causes a monotone fitness decline of **~0.7–3.5%** under the median posterior, with the upper CI reaching **~30%** if the true lag is near +4 °C. Per-decade rate under SSP2-4.5 ≈ **0.45 percentage points per decade** at the aggregate typical-plant level.

bioRxiv preprint doi: https://doi.org/10.64898/2026.06.02.729671; this version posted June 3, 2026. The copyright holder for this preprint (which was not certified by peer review) is the author/funder, who has granted bioRxiv a license to display the preprint in perpetuity. It is made available under a CC-BY 4.0 International license.

Table S5. Potential fitness losses of each species.
Assuming correct inference of lag of course, and providing

Species	μ_λ (°C)	V_s (°C^2)	Baseline today	SSP1-1.9	SSP1-2.6	SSP2-4.5	SSP3-7.0	SSP5-8.5
Arabidoss thaliana	+1.91	815	0.2%	0.4%	0.5%	0.6%	0.8%	1.0%
*Quercus lobata**	+6.00	~30	**45%**	**51%**	**55%**	**59%**	**63%**	**67%**
Panicum virgatum	+6.10	110	**16%**	**18%**	**20%**	**22%**	**24%**	**26%**
Fagus sylvatica	+2.60	1,057	0.3%	0.5%	0.6%	0.7%	0.9%	1.0%
Picea glauca	−0.80	70	0.5%	0.1%	0.0%	0.2%	0.6%	1.2%
Plantago lanceolata	−4.30	353	2.6%	2.0%	1.6%	1.3%	1.0%	0.7%
Juglans nigra	+0.50	44	0.3%	1.1%	2.2%	3.6%	5.4%	7.4%
Silene floscuculi	−6.50	69	**26%**	**23%**	**20%**	**18%**	**15%**	**13%**
Chamaeristasta fasciculata	−3.20	127	4.0%	2.8%	2.1%	1.4%	0.9%	0.5%
Boechera stricta	−1.50	8	**13%**	6.1%	2.2%	0.2%	0.2%	2.2%
Lotus corniculatus	−1.40	8	**12%**	4.9%	1.6%	0.1%	0.6%	3.0%
Populus tremuloides	−0.50	27	0.5%	0.0%	0.3%	1.2%	2.6%	4.6%
Pinus pinaster	+6.00	244	7.1%	8.3%	9.3%	**10.3%**	**11.4%**	**12.6%**
Chamaecyparis thyoides	−1.30	56	1.5%	0.6%	0.1%	0.0%	0.1%	0.6%
A. lyrata ssp. *petraea*	−3.70	127	5.2%	4.0%	3.0%	2.2%	1.6%	1.0%
Pinus canariensis	+5.00	155	7.7%	9.3%	**10.6%**	**12.0%**	**13.5%**	**15.0%**
Erythranthe guttata	+2.20	14	**16%**	**23%**	**29%**	**35%**	**42%**	**48%**

* *Quercus lobata* V_s is a placeholder (our fit gave $\beta_2 > 0$, not identifiable); value assumes Browne's among-source summer-Tmax SD ≈ 2.5–3.5 °C and μ_λ ≈ +6 °C lower bound.

Table S6. Cumulative aggregate of fitness decline (2050 land warming)

Summing across the 17-species panel:

Scenario	Cumulative decline (Σ species, %)	Mean per-species decline (%)
SSP1-1.9	**154**	9.1
SSP1-2.6	**158**	9.3
SSP2-4.5	**168**	9.9
SSP3-7.0	**184**	10.8
SSP5-8.5	**205**	**12.1**

bioRxiv preprint doi: https://doi.org/10.64898/2026.06.02.729671; this version posted June 3, 2026. The copyright holder for this preprint (which was not certified by peer review) is the author/funder, who has granted bioRxiv a license to display the preprint in perpetuity. It is made available under a CC-BY 4.0 International license.

Table S7. Curiosity testing lag measurements based on accession or garden characteristics.

Scenario-type	Scenario	mean λ [95% CI]	Observation size
Experimental	Garden located in non-native range	1.8[0.5:2.9]	7295
Experimental	Garden located in native range	-1.8[-2.7:-0.9]	20372
Experimental	Experiment planted in ground	-6.1[-29.6:-1.3]	1199
Experimental	Experiment planted in pots	-1.8[-2.9:-0.9]	26468
Experimental	Plants started outside as seedlings	-1.6[-4.2:0]	2460
Experimental	Plants stared outsides as seeds	-2[-3.2:-0.8]	25207
Experimental	Water was added to experimental design	-38.9[-159.5:82.2]	6879
Experimental	No water was added to experimental design	-1.9[-3:-0.8]	23548
Population	Mediterranean population	-11.1[-25.2:-1.5]	13042
Population	Non-mediterranean population	82.5[-242.1:299.3]	14625
Population	Population collected before year 2000	-0.8[-2.7:0.5]	9887
Population	Populations collected after year 2000	-2.7[-4.4:-1.3]	17780
Population	Population present in 2 or more gardens	-2.5[-3.7:-1.5]	26192
Population	Population present in only 1 garden	0.6[-6.8:5.9]	1475
Population	Population from non-native range	2.8[-6.1:21.1]	607
Population	Population from native range	-1[-2:-0.2]	27060
Population	Populations are southern relicts	-1.9[-5:0.4]	1134
Population	Populations are not southern relicts	-2.4[-3.8:-1.4]	26533

Table S8. Sensitivity testing, leave one out and keep one in results.

Sensitivity type	Study	mean λ [95% CI]
Leave-one-out	Brachi_2015	-1.50[-2.57:-0.75]
Leave-one-out	Expositoalonso_2018	-1.49[-2.39:-0.65]
Leave-one-out	Expositoalonso_2019	-1.24[-2.11:-0.51]
Leave-one-out	Korves_2007	-1.46[-2.32:-0.71]
Leave-one-out	Leventhal_2025	-1.27[-2.21:-0.20]
Leave-one-out	Manzanopiedras_2014	-1.65[-2.38:-0.84]
Leave-one-out	Rutter_2007	-1.50[-2.55:-0.51]
Leave-one-out	Singh_2017	-1.50[-2.28:-0.73]
Leave-one-out	Wilczek_2014	-1.30[-2.30:-0.39]
Leave-one-out	Wu_2026	-10.86[-35.44:-3.50]
Keep-one-in	Brachi_2015	0.62[-23.16:20.12]
Keep-one-in	Expositoalonso_2018	6.03[-35.37:50.79]
Keep-one-in	Expositoalonso_2019	5.87[-177.63:202.62]
Keep-one-in	Korves_2007	0.31[-29.67:25.22]
Keep-one-in	Leventhal_2025	-10.32[-106.59:92.04]
Keep-one-in	Manzanopiedras_2014	-8.44[-53.20:52.94]
Keep-one-in	Rutter_2007	-5.80[-5.24:8.19]
Keep-one-in	Wilczek_2014	-6.26[-22.12:-1.28]
Keep-one-in	Wu_2026	-1.14[-2.24:-0.14]

bioRxiv preprint doi: https://doi.org/10.64898/2026.06.02.729671; this version posted June 3, 2026. The copyright holder for this preprint (which was not certified by peer review) is the author/funder, who has granted bioRxiv a license to display the preprint in perpetuity. It is made available under a CC-BY 4.0 International license.

Supplemental Figures

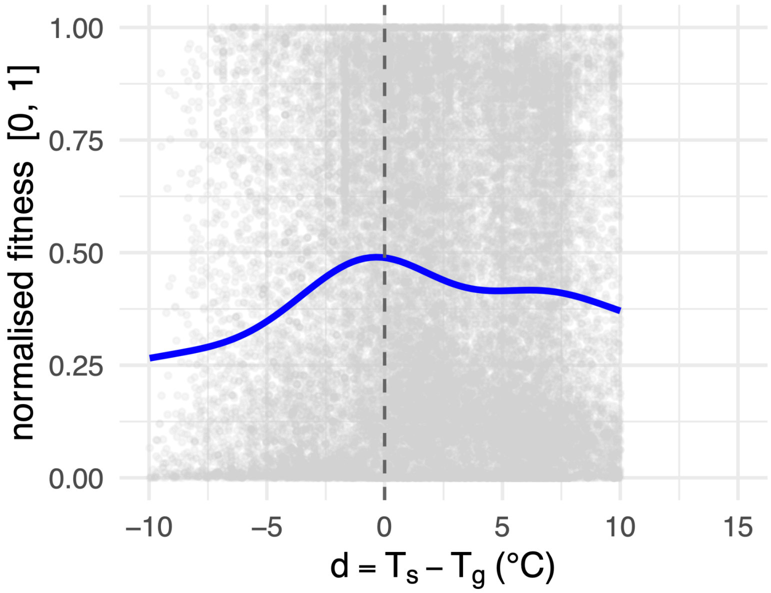

Figure S1. Relationship between fitness and annual temperature mismatch between experimental and genotype home site.
Each point shows the normalised fitness of a population in a common garden as a function of d-space. The blue curve shows a smoothed fit (GAM) with 95% point-wise uncertainty omitted for clearing viewing of the raw fitness points. The vertical dashed line means no mismatch in temperature ($d = 0$),

bioRxiv preprint doi: https://doi.org/10.64898/2026.06.02.729671; this version posted June 3, 2026. The copyright holder for this preprint (which was not certified by peer review) is the author/funder, who has granted bioRxiv a license to display the preprint in perpetuity. It is made available under a CC-BY 4.0 International license.

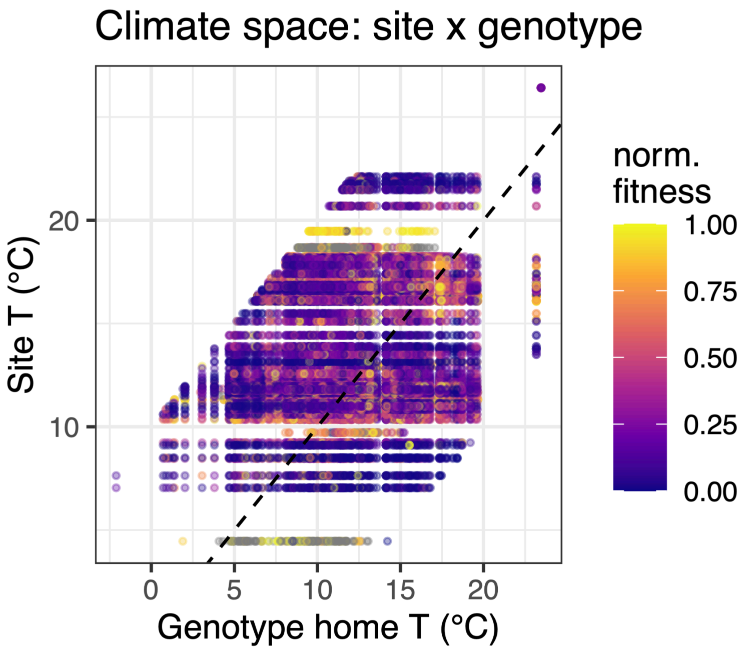

Figure S2. Climate space of common garden sites and genotypes.
Each point represents a genotype-by-site combination, plotted by the long-term mean temperature at the genotype's home site (x-axis) and the common garden site (y-axis), with point color indicating normalized fitness. The dashed 1-1 line denotes perfect temperature matching between home and garden climates, illustrating that some of the genotypes we used are well-matched while others are not well-matched to their experimental site.

bioRxiv preprint doi: https://doi.org/10.64898/2026.06.02.729671; this version posted June 3, 2026. The copyright holder for this preprint (which was not certified by peer review) is the author/funder, who has granted bioRxiv a license to display the preprint in perpetuity. It is made available under a CC-BY 4.0 International license.

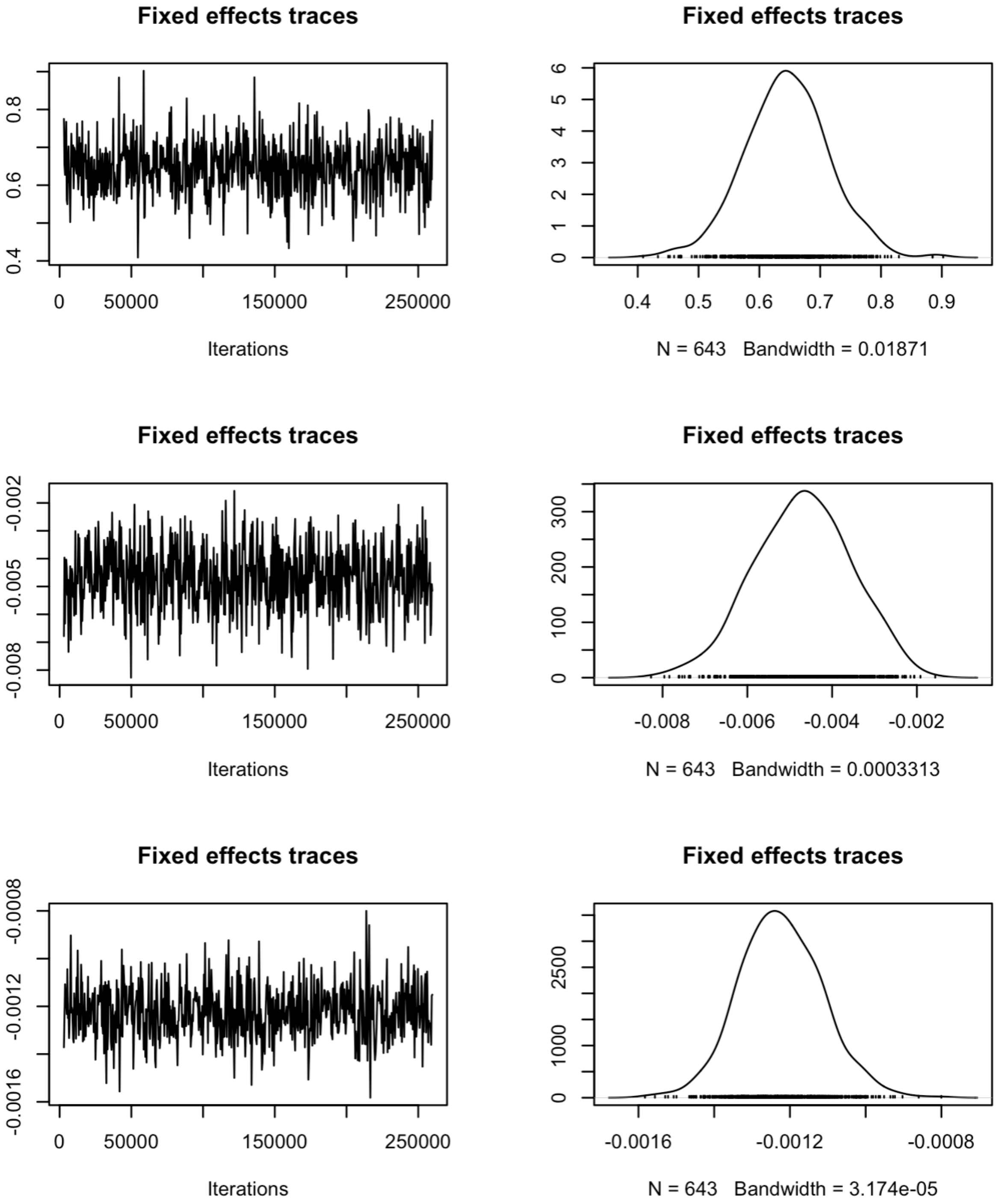

Figure S3. Model diagnostics for fixed effects in the adaptation-lag model.
Trace plots and marginal posterior densities for fixed effects indicate good mixing and approximate convergence of the MCMC chains.

bioRxiv preprint doi: https://doi.org/10.64898/2026.06.02.729671; this version posted June 3, 2026. The copyright holder for this preprint (which was not certified by peer review) is the author/funder, who has granted bioRxiv a license to display the preprint in perpetuity. It is made available under a CC-BY 4.0 International license.

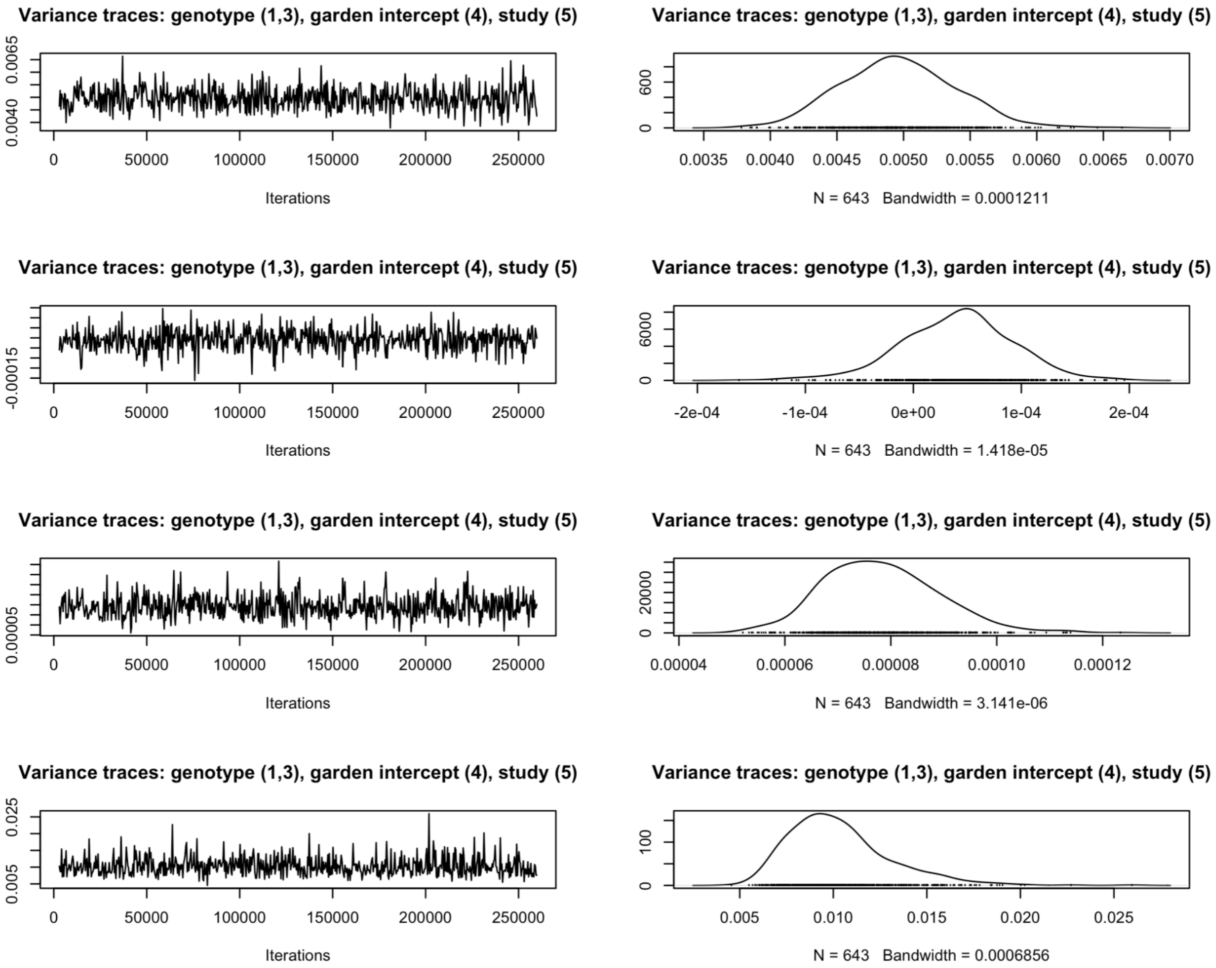

Figure S4. MCMC diagnostics for variance components in the adaptation-lag model. Trace plots and marginal posterior densities for the genotype, garden (site) intercept, and study-level random effect variances indicate good mixing and approximate convergence of the MCMC chains.

bioRxiv preprint doi: https://doi.org/10.64898/2026.06.02.729671; this version posted June 3, 2026. The copyright holder for this preprint (which was not certified by peer review) is the author/funder, who has granted bioRxiv a license to display the preprint in perpetuity. It is made available under a CC-BY 4.0 International license.

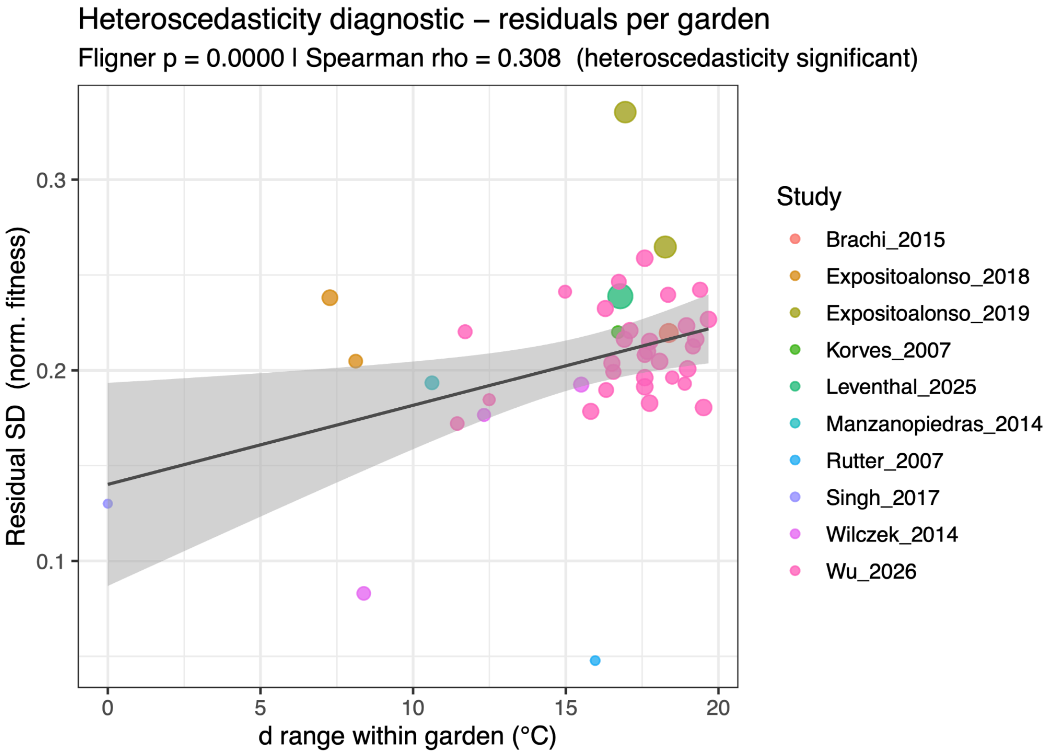

Figure S5. Heteroscedasticity diagnostics for the adaptation-lag model. Each point shows the standard deviation of model residuals (normalized fitness) with a garden plotted against the range of temperature mismatch *d* experienced in that garden, with colors indicating study identity and a fitted linear trend line illustrating increasing residual variance with increasing *d* range.

bioRxiv preprint doi: https://doi.org/10.64898/2026.06.02.729671; this version posted June 3, 2026. The copyright holder for this preprint (which was not certified by peer review) is the author/funder, who has granted bioRxiv a license to display the preprint in perpetuity. It is made available under a CC-BY 4.0 International license.

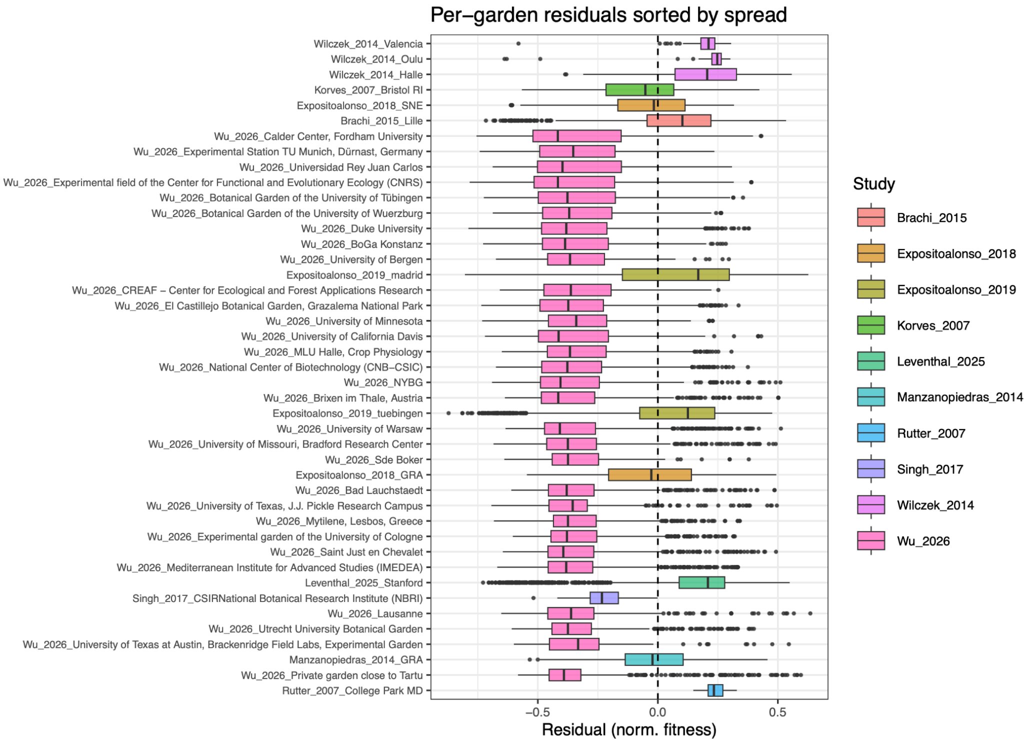

Figure S6. Heteroscedasticity diagnostics showing per-garden distribution of residuals from the adaptation-lag model.

Boxplots show the distribution of normalized fitness residuals for each common garden, ordered by residual spread, with colors indicating study identity and the dashed vertical line marking zero residual (perfect model fit).

bioRxiv preprint doi: https://doi.org/10.64898/2026.06.02.729671; this version posted June 3, 2026. The copyright holder for this preprint (which was not certified by peer review) is the author/funder, who has granted bioRxiv a license to display the preprint in perpetuity. It is made available under a CC-BY 4.0 International license.

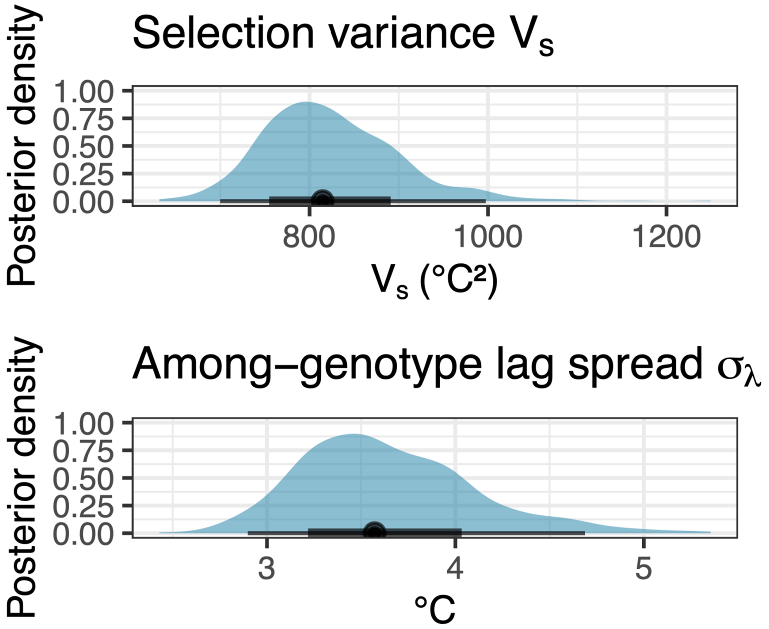

Figure S7. Posterior distributions for key adaptation-lag parameters.

Shaded densities show the posterior for the selection and variance V_s (top; units °C^2) and the among genotype lag spread σ_λ (bottom; units °C^2), with horizontal bars indicating central credible intervals and points marking posterior medians.
